# Full-Length Transcriptome Analyses of Genes Involved in Triterpenoid Saponin Biosynthesis of *Psammosilene tunicoides* Hairy Root Cultures With Exogenous Salicylic Acid

**DOI:** 10.3389/fgene.2021.657060

**Published:** 2021-03-29

**Authors:** Lingye Su, Shufang Li, Hanhan Qiu, Hongfeng Wang, Congcong Wang, Chunmei He, Mingfeng Xu, Zongshen Zhang

**Affiliations:** ^1^Guangdong Provincial Key Laboratory of Silviculture Protection and Utilization, Guangdong Academy of Forestry, Guangzhou, China; ^2^School of Biology Engineering, Dalian Polytechnic University, Dalian, China

**Keywords:** salicylic acid, triterpenoid saponins, *Psammosilene Tunicoides*, full-length transcriptome, hairy root

## Abstract

Triterpenoid saponins constitute a diverse class of bioactive compounds in medicinal plants. Salicylic acid (SA) is an efficient elicitor for secondary metabolite production, but a transcriptome-wide regulatory network of SA-promoted triterpenoid saponin biosynthesis remains little understood. In the current study, we described the establishment of the hairy root culture system for *Psammosilene tunicoides*, a triterpenoid saponin-producing medicinal herb in China, using genetic transformation by *Agrobacterium rhizogenes*. Compared to controls, we found that total saponin content was dramatically increased (up to 2.49-fold) by the addition of 5 mg/L SA in hairy roots for 1 day. A combination of single-molecule real-time (SMRT) and next-generation sequencing (Illumina RNA-seq) was generated to analyze the full-length transcriptome data for *P. tunicoides*, as well as the transcript profiles in treated (8 and 24 h) and non-treated (0 h) groups with 5 mg/L SA in hairy roots. A total of 430,117 circular consensus sequence (CCS) reads, 16,375 unigenes and 4,678 long non-coding RNAs (lncRNAs) were obtained. The average length of unigenes (2,776 bp) was much higher in full-length transcriptome than that derived from single RNA-seq (1,457 bp). The differentially expressed genes (DEGs) were mainly enriched in the metabolic process. SA up-regulated the unigenes encoding SA-binding proteins and antioxidant enzymes in comparison with controls. Additionally, we identified 89 full-length transcripts encoding enzymes putatively involved in saponin biosynthesis. The candidate transcription factors (WRKY, NAC) and structural genes (*AACT*, *DXS*, *SE*, *CYP72A*) might be the key regulators in SA-elicited saponin accumulation. Their expression was further validated by quantitative real-time PCR (qRT-PCR). These findings preliminarily elucidate the regulatory mechanisms of SA on triterpenoid saponin biosynthesis in the transcriptomic level, laying a foundation for SA-elicited saponin augmentation in *P. tunicoides*.

## Introduction

Triterpenoid saponins constitute a diverse class of natural products, and their biosynthesis is responsible for the pharmacological properties of numerous traditional medicinal plants (Moses et al., 2013). The *Caryophyllaceae* family is chemically characterized by high production of triterpenoid saponins (Cheikh-Ali et al., 2019). *Psammosilene tunicoides* WC Wu et CY Wu, a monotypic species of *Caryophyllaceae*, has been commonly used as a valuable traditional medicine in China (Wang and Zhang, 1992). This medicinal plant has various therapeutic properties including pain-relief, haemostasia, anti-inflammation and immunomodulation (Zhao et al., 2011; Zhang et al., 2012). The tuberous roots of *P. tunicoides* (named “Jintiesuo“) constitute an important ingredient in a famous traditional Chinese medicine “Yunnan Baiyao“ (Li et al., 2011; Zhao et al., 2011). Previous phytochemical and pharmacological analyses have revealed that the oleanane-type triterpenoid saponins constitute the essential bioactive components in *P. tunicoides* (Zhong et al., 2002; Deng et al., 2009; Wen et al., 2020). However, *P. tunicoides* is only distributed in southwest China, and it is a slow-growing plant with limited saponin production. Due to the high market demand and overexploitation, the natural resources of *P. tunicoides* have dwindled and become endangered. Thus, it has been listed in the National Key Protected Plants in China. As an alternative to wild exploitation, hairy root cultures using genetic transformation by *Agrobacterium rhizogenes* have been developed for sustainable production of the bioactive components (Gutierrez-Valdes et al., 2020). Moreover, these culture systems were also effectively used in elucidating the biosynthesis pathway of bioactive molecules, such as in *Isatis indigotica* (Chen et al., 2015) and *Salvia miltiorrhiza* (Zhou et al., 2017). Hence, this approach provides the foundation for *in vitro* triterpenoid saponin production by synthetic biology strategies in *P. tunicoides*.

In general, terpenoids are regarded as phytoanticipins, which are involved in responding to biotic and abiotic stresses (Dixon, 2001). Salicylic acid (SA) is an endogenous signal substance that exists universally in plants (Wu et al., 2019). It exhibit that SA is perceived by the Non-expresser of PR (NPRs) receptors, and functions directly in the activation of defense responses (Sawai and Saito, 2011). Additionally, SA participates in promoting the yields of secondary metabolites (including terpenoids) in medicinal plants (Yendo et al., 2010; Ramirez-Estrada et al., 2016), and functions as one of the most important elicitors in hairy root cultures (Gutierrez-Valdes et al., 2020). A comprehensive understanding of SA-mediated regulatory networks has been widely elucidated using transcriptome analysis in plants. However, these studies mainly focused on the molecular mechanism of SA in stress response (Kim et al., 2013; Fan et al., 2017), with minimal focus on the elicitation of terpenoid biosynthesis. To date, the relationship between SA elicitors and terpenoid accumulation is unclear in *P. tunicoides*. Transcriptome-wide analysis of the SA-elicited regulatory chain may reveal the biosynthesis pathway of terpenoids in this species.

Previous studies attempted to elucidate biosynthesis pathways of oleanane-type triterpenoid saponins, such as soyasaponin and glycyrrhizin (Sawai and Saito, 2011; Moses et al., 2014). In general, three stages were reported to be involved in the triterpenoid saponin biosynthesis process: terpene precursor biosynthesis, triterpenoid skeleton biosynthesis, and saponin structural diversification (Moses et al., 2014). The first stage generates two types of terpene precursors, isopentenyl pyrophosphate (IPP) and dimethylallyl-pyrophosphate (DMAPP), through the mevalonate (MVA) and 2-C-methyl-derythritol-4-phosphate (MEP) pathways, respectively. These two precursors are then converted to β-amyrin to form a skeleton of triterpenoid compounds, and further generate various oleanane-type triterpenoid saponins by site-specific oxidization and glycosylation (Moses et al., 2014; Seki et al., 2015). These processes are essentially catalyzed by diverse rate-limiting biosynthetic enzymes, which are encoded by multi-gene families (Moses et al., 2013). In addition, transcription factors (TFs), such as WRKY, NAC and MYB, modulate downstream gene expression associated with related enzymes, and thus are essential in transcriptional regulation of secondary metabolism (Endt et al., 2002).

Next-generation sequencing (e.g., Illumina RNA-seq) is an effective method to provide transcriptome-wide analysis of sequence data and differentially expressed genes (DEGs), especially for non-model plant species (Ma et al., 2016; Choudhri et al., 2018). RNA-seq has been performed to identify the putative genes involved in terpenoid biosynthesis in multiple plants (Luo et al., 2011; Ma et al., 2016; Wang et al., 2017; Zhou et al., 2017; Choudhri et al., 2018; Shan et al., 2020). Nevertheless, the short read lengths from RNA-seq restrict the construction of completely assembled transcripts, making it difficult to obtain full-length sequences (Wu et al., 2019, 2020). As a third-generation sequencing technique, single-molecule real-time (SMRT) sequencing can generate full-length cDNA sequences without post-sequencing assembly, which basically overcomes the limitation of RNA-seq (Wu et al., 2019, 2020). Thus, SMRT sequencing could provide an accurate technique for gene annotation, novel gene discovery and long non-coding RNA (lncRNA) identification. To eliminate its high error rate, SMRT sequencing still needs to be corrected with RNA-Seq reads. A combined analysis of SMRT sequencing and RNA-Seq is a suitable approach to obtain high-quality transcripts, which has been recently used in a variety of traditional Chinese medicinal plants like *Pogostemon cablin* (Chen et al., 2019) and *Coptis deltoidea* (Zhong et al., 2020). To date, although several genes have been obtained and cloned in *P. tunicoides* from RNA-seq (Jiang and Qian, 2014; Zhang et al., 2018), a high-quality transcriptome data is still lack in *P. tunicoides*.

We previously established a high-efficiency *P. tunicoides* hairy root culture (PTHRC) system (Wang et al., 2015). Here, the effects of SA elicitors on triterpenoid saponin production were determined in PTHRCs. The full-length transcriptome of *P. tunicoides* was generated for the first time using SMRT sequencing, corrected by RNA-Seq at different stages of SA treatments. Functional annotation and lncRNA identification were performed. DEGs related to SA-elicited saponin biosynthesis were identified on the transcriptome, and verified by quantitative real-time PCR (qRT-PCR). The regulatory chain of “signaling cascade-TF regulation-enzymatic catalysis” was then illustrated. High-quality transcriptome data were obtained, which can be used to elucidate the regulatory mechanisms of SA in saponin biosynthesis in PTHRCs, and provide a theoretical basis for effectively improving saponin production in *P. tunicoides*.

## Materials and Methods

### Plant Materials

PTHRCs were established as reported in our previous study (Wang et al., 2015). Briefly, sterile healthy *P. tunicoides* plants were maintained on MS media supplemented with 20 g/L sucrose (pH 5.8) at 25 °C and a 16-h photoperiod. Excised leaves of *P. tunicoides* were used to induce hairy roots by infection with *A. tumefaciens* strain ATCC15834. The hairy roots were routinely subcultured and maintained in 100 mL liquid MS media supplemented with 30 g/L sucrose (pH 5.8) in conical flasks (250 mL) in a shaker with a speed of 100 rpm for 35 days. The growth conditions for suspension cultivations were 25 ± 1°C in darkness.

### Elicitor Preparation and Addition

SA (Sigma-Aldrich, United States) was dissolved in ultrapure water as a stock solution (10 mg/mL) and filter-sterilized. After 35 days of suspension cultivation, hairy roots at their maximum weight were used to perform an SA elicitation experiment. The subcultured media were renewed by 100 mL fresh liquid media supplemented with 30 g/L sucrose (pH 5.8), in which the SA stock solution was added to give the final concentrations of 5, 10, 20 mg/L SA. The same volume of ultrapure water was added as control (CK) cultures.

### Saponin Extraction and Quantification

The treated (5, 10, 20 mg/L SA) and CK hairy root groups were sampled at 1, 3, 5, 7, and 9 days for saponin extraction and quantification. All experiments were repeated at least three times. The samples were vacuum freeze-dried for 12 h (Telstar, Spain), and ground into powders. The powders (1 g) were extracted using 80% ethanol with ultrasonication for 100 min. After extraction with 40 mL of n-butanol three times, the combined extracts were rotary evaporated at 40 °C (BUCHI, Switzerland), and redissolved in 10 mL of methanol.

Total saponin content was determined by vanillin-perchloric acid colorimetry according to a previous study (Xiang et al., 2001), and oleanolic acid was used as the reference standard. Levels of two representative saponin derivatives [quillaic acid (QA) and gypsogenin (Gyp)] were quantified by high-performance liquid chromatography (HPLC) based on a previous study (Kolodziej et al., 2018). Chromatographic analysis was carried out on a Shimadzu series LC-20 AD XR instrument, with an SPD-M20A diode array detector, on a reverse-phase Sunniest C18 (ChromaNik, Japan) analytical column (250 mm × 4.6 mm, 5 μm) at 25°C. The mobile phase consisted of acetonitrile (solvent A) and 0.1% (v/v) H_3_PO_4_ aqueous solution (solvent B) using the gradient elution as follows: 50% A 0–2 min, 50–100% A 2–35 min, 100% A 35–40 min. The injection volume was 10 μL. The detection wavelength was 210 nm, and the flow rate was 1 mL/min. Gyp and QA standards (Yuanye, China) were used as standard calibration curves.

### RNA Sample Preparation

For transcriptome sequencing, antioxidant enzyme and qRT-PCR analysis, the 35-day-old hairy roots were harvested from 5 mg/L SA treatment at 8 (SA_8 h) and 24 h (SA_24 h) after initiating treatments. Untreated hairy roots were collected before SA treatment, and indicated as controls (SA_0 h). Total RNA was extracted using TRIzol reagent (Invitrogen, United States) according to the manufacturer’s instructions. RNA purity was checked using the NanoPhotometer^®^ spectrophotometer (IMPLEN, United States). RNA concentration was assessed using a Qubit^®^ RNA Assay Kit in a Qubit^®^ 2.0 Fluorometer (Life Technologies, United States).

### PacBio SMRT Library Preparation and Sequencing

Equal amounts of RNAs from different hairy root groups (SA_0h, SA_8h, and SA_24h) were pooled to provide the total RNA of *P. tunicoides*. The mRNA was enriched using the Oligo d(T) magnetic beads, then reverse transcribed to cDNA using Clontech SMARTer PCR cDNA Synthesis Kit (Clontech, United States). Amplification of double-stranded cDNA was followed by size selection using the BluePippin system (Sage Science, United States), and fragments of 1–6 kb were retained. The full-length cDNA was generated, and the cDNA ends were repaired and ligated to sequencing adapters. SMRTbell template libraries were obtained and subsequently sequenced on the PacBio Sequel RS sequencing instrument.

### SMRT Read Processing

Raw sequence data obtained from SMRT sequencing were processed using SMRTlink6.0 software (Chin et al., 2013). Circular consensus sequence (CCS) reads were generated from subread BAM files, and classified into full-length non chimera (FLNC) reads, full-length chimeric reads, non–full-length (NFL) reads, and short reads based on poly(A) signal, 5’ and 3’ adaptor check. NFL and full-length FASTA files were then fed into the cluster consensus isoforms by isoform-level clustering (ICE) (Gordon et al., 2015). Finally, the redundancies were removed using CD-HIT-EST to obtain unigenes (Huang et al., 2010).

### Illumina Library Construction and Sequencing

RNA samples from three SA-treated hairy roots (SA_0h, SA_8h and SA_24h) were used for Illumina library construction and sequencing. Each group had three biological replicates. RNA-seq libraries were generated using NEBNext^®^ Ultra^TM^ RNA Library Prep Kit for Illumina^®^ (NEB, United States) following the manufacturer’s recommendations and index codes were added to attribute sequences of each sample. The libraries were constructed with insert sizes of 250–300 bp in length. Clustering of the index-coded samples was performed on a cBot Cluster Generation System using TruSeq PE Cluster Kit v3-cBot-HS (Illumina). After cluster generation, the libraries were sequenced on an Illumina Hiseq Xten platform from Novogene Experimental Department (Beijing, China) and the raw data were generated. The clean data were obtained by removing reads containing adapters, poly-N and low quality reads from raw data using Trimomatic (v0.36) (Bolger et al., 2014). The Q20, Q30 and GC-content of the clean data were calculated. The raw sequence data have been deposited in the Genome Sequence Archive^1^ under accession number CRA002795.

### Gene Function Annotations and Classifications

The gene function was annotated, and classifications were based on NCBI non-redundant protein sequences (Nr)^2^, NCBI nucleotide sequences (Nt; see text footnote 1), protein family (Pfam)^3^, eukaryotic/clusters of orthologous groups (KOG/COG)^4^, Swiss-Prot^5^, Kyoto Encyclopedia of Genes and Genomes (KEGG)^6^ and Gene Ontology (GO)^7^ databases with local BLAST programs (*E*-value *<* 1.0E^–5^). Hmmscan was adopted for Pfam annotation^8^, and Blast2GO was used for GO annotation (Conesa et al., 2005). The ANGEL software was used to predict coding sequences (CDS)^9^. The iTAK software was applied to identify the TF family database (Zheng et al., 2016).

### Differential Gene Expression Analysis

The gene expression levels were determined by RSEM software with bowtie2 (mismatch 0) (Li and Dewey, 2011). Illumina clean data were mapped onto the SMRT sequencing data, and the readcount for each gene was obtained from the mapping results. Then the unique readcounts for each transcript were normalized by calculating fragment per kilobase per million (FPKM). DEGs of different libraries were analyzed using the DESeq R package (1.10.1) (Anders and Huber, 2010). Genes with an adjusted *P*-value < 0.05 and | log2FoldChange| > 1 found by DESeq were assigned as differentially expressed. GO enrichment analysis of the DEGs was implemented by the GOseq R packages based Wallenius non-central hypergeometric distribution (Young et al., 2010). KEGG enrichment analysis of DEGs was done using KOBAS software (Xie et al., 2011). Heatmaps were drawn by TBtools software (Chen et al., 2020). A phylogenetic tree was generated by the neighbor-joining method in MEGA7 (1,000 bootstrap replicates) (Kumar et al., 2016) and iTOL v5^10^. The protein sequences were obtained from UniProt database^11^.

### Determination of Antioxidant Enzyme Activity

Hairy root samples were fully ground into 0.2 M phosphate buffer solution (pH 7.4) at 4°C, then the homogenates were centrifuged at 12,000 rpm at 4°C for 10 min. The supernatants were collected for peroxidase (POD) and glutathione reductase (GR) activities measurement according to the instructions of the assay kits (Nanjing Jiancheng, China).

### Validation of DEGs With qRT-PCR Analysis

Total RNA was extracted using RNAprep Pure Plant Kit (Tiangen, China) according to the manufacturer’s protocol. cDNA was obtained from 1 μg of total RNA using a TransScript One-Step gDNA Removal and cDNA Synthesis SuperMix (TransGen Biotech, China). qRT-PCR assays were performed using SuperReal PreMix Plus kit (Tiangen, China) with SYBR Green method on an ABI 7500 Real-Time PCR System (Thermo Fisher Scientific, United States). The *Ptβ-Actin* was used as the reference gene to normalize the relative expression levels via 2^–ΔΔCt^ method (Li et al., 2016). All qRT-PCR experiments were performed in three biological replicates. The primers were designed using Primer Premier 5.0 (Lalitha, 2000), and listed in Supplementary Table 1.

## Results

### SA Effects on Saponin Accumulation in PTHRCs

**FIGURE 1:**
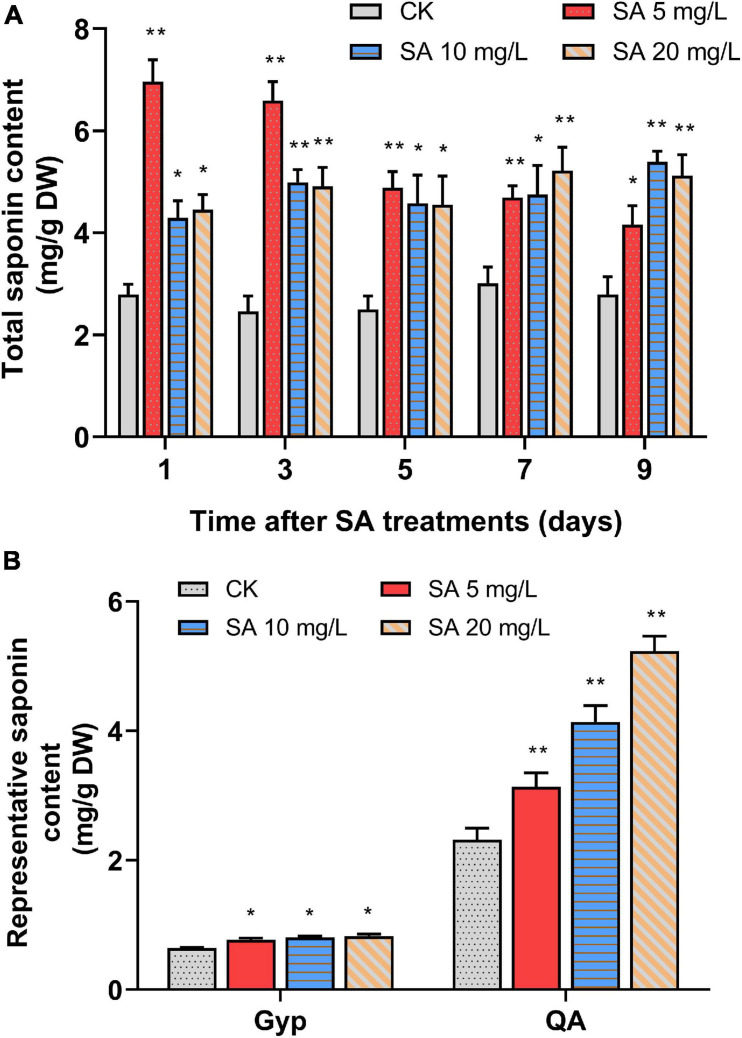
Effects of SA elicitors on triterpenoid saponin production in *P. tunicoides* hairy root cultures. **(A)** The total saponin productions in hairy root cultures of control (CK) and elicited groups by 5, 10, and 20 mg/L SA for 1, 3, 5, 7, and 9 days. **(B)** The gypsogenin (Gyp) and quillaic acid (QA) contents in hairy root cultures of CK and elicited groups by 5, 10, and 20 mg/L SA for 1 day. The values represent the mean value ± SE from three biological replicates. *, ** Indicate significant differences in comparison with values of CK at *P* < 0.05 and *P* < 0.01 level (*t*-test), respectively.

To investigate the elicitation effects of SA on triterpenoid saponin accumulation, 35-day-old *P. tunicoides* hairy roots were treated with a series of concentrations (5, 10, 20 mg/L) of SA, then cultivated continuously for 9 days. In comparison with the CK, the total saponin levels of PTHRCs were significantly increased by all groups of SA elicitation (Figure 1A). The treatments with 5 mg/L SA were most efficient, triggering the total saponin content of PTHRCs to peak at 2.49-fold higher than that of controls after 1 day (6.96 ± 0.43 mg/g DW). Hence, SA acted as an effective elicitor to positively modulate triterpenoid saponin production in PTHRCs, and 5 mg/L SA might be the optimal concentration for further analysis.

Previous studies illuminated that the molecular structures of *P. tunicoides* saponins were dominated by two representative aglycones: QA and Gyp (Cheikh-Ali et al., 2019). Furthermore, the trends of QA and Gyp with 1 day of SA treatments were also considered in acidolysis extraction of PTHRCs using HPLC (Supplementary Figure 1). Compared with control, levels of QA (3.14 ± 0.21 mg/g DW to 5.23 ± 0.23 mg/g DW) and Gyp (0.77 ± 0.02 mg/g DW to 0.83 ± 0.03 mg/g DW) in SA-elicited PTHRCs (5, 10 or 20 mg/L SA for 1 day) were increased by 35.4–125.9% and 20.1–28.4%, respectively (Figure 1B). Taken together, SA promoted yields of the representative saponin aglycones in PTHRCs in a relatively short time.

### Full-Length Transcriptome Analysis

**FIGURE 2:**
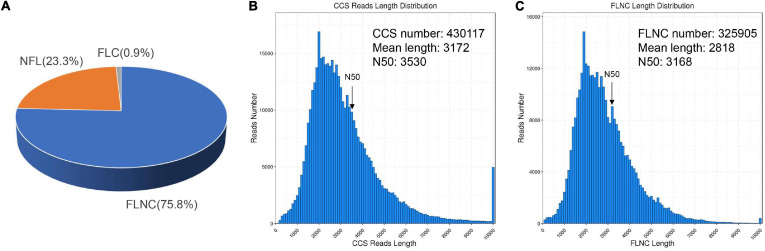
Summary of PacBio SMRT sequencing. **(A)** The classification of circular consensus sequence (CCS) reads in SMRT sequencing of *P. tunicoides* hairy root cultures. The percentage of full-length non chimera (FLNC), non–FL (NFL) and full-length chimera (FLC) reads. **(B,C)** The number and length distributions of CCS **(B)** and FLNC **(C)** reads.

To investigate how SA triggered the transcript pathway of triterpenoid saponin biosynthesis in PTHRCs, we conducted transcriptome analysis using combined Illumina- and PacBio SMRT-based sequencing. Nine RNA samples from the different time points of SA treatments (0, 8, and 24 h) were sequenced using the Illumina Hiseq Xten platform. A total of 487,691,344 raw reads ranging from 48 to 62 million for each sample and 475,534,604 clean reads (97.5% of raw reads) ranging from 47 to 61 million for each sample were obtained. The quality of clean reads was good with Q20 > 96%, Q30 > 91.8% (except SA_0h_3) and GC 42–44% (Supplementary Table 2). To obtain wide coverage of the *P. tunicoides* transcriptome, pooled samples from different time points of SA treatments (0, 8, and 24 h) were sequenced using the PacBio RS II platform. A total of 39.43 Gb raw reads and 15,034,708 subreads were yielded. Then, 430,117 CCS reads were obtained after processing raw sequencing data, including 325,905 (75.8%) FLNC reads, and 100,217 (23.3%) NFL reads (Figure 2A). The CCS reads contained a mean length of 3,172 bp with N50 of 3,530 bp, while FLNC reads showed an average length of 2,818 bp with N50 of 3,168 bp (Figures 2B,C). After correcting with Illumina reads and removing the redundant sequences via CD-Hit analysis, 35,262 non-redundant transcript isoforms were generated.

We found that the unigene number obtained from the PacBio transcriptome (16,375) was much lower than that of the Illumina transcriptome (182,277) (Table 1). However, PacBio unigenes showed a significantly longer average length and larger N50 values when compared with Illumina unigenes (Table 1). We found that nearly 26.4% of Illumina unigenes had a length of < 500 bp; whereas only 0.3% of PacBio unigenes had lengths < 500 bp, and approximately 96.9% genes from SMRT sequencing were over 1,000 bp (Table 1). In addition, 65.1% of unigenes from the PacBio platform had complete CDSs, while only 13.5% of unigenes in the Illumina transcriptome had full-length CDSs (Table 1).

**TABLE 1:** Comparison of unigene information from Illumina and Pacbio platform.

		Illumina transcriptome data	Pacbio transcriptome data
**Unigene assembly**			
Total numbers		182,277	16,375
Distribution	<500 bp	48,079 (26.4%)	55 (3.3%)
	500–1 kbp	41,975 (23.0%)	453 (2.8%)
	1 k–2 kbp	45,644 (25.0%)	4,573 (27.9%)
	>2 kbp	46,579 (25.6%)	11,294 (69.0%)
Length	Average	1,457	2,776
	N50	2,277	3,131
	N90	653	1,731
**CDS prediction**			
Total numbers		182,814	16,393
Type	Complete	24,639 (13.5%)	10,680 (65.1%)
	5′ partial	12,612 (6.9%)	1,964 (12.0%)
	3′ partial	123,358 (67.5%)	53 (0.3%)
	No affirmed	22,205 (12.1%)	3,696 (22.5%)

### Functional Annotation and Categorization

**FIGURE 3:**
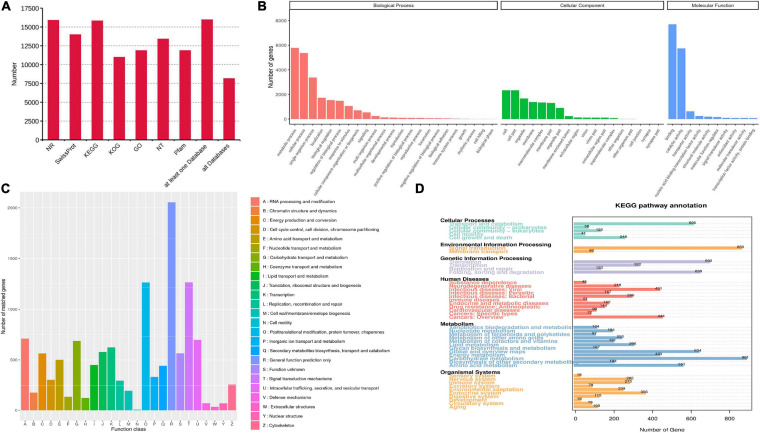
Functional annotation and classification of transcriptomes. **(A)** The unigene numbers annotated in non-redundant protein sequences (Nr), NCBI nucleotide sequences (Nt), protein family (Pfam), eukaryotic/clusters of orthologous groups (KOG/COG), Swiss-Prot, Kyoto Encyclopedia of Genes and Genomes (KEGG) and Gene Ontology (GO) databases. **(B–D)** The pathway enrichment analysis based on GO **(B)**, KOG **(C)**, and KEGG **(D)** databases.

Seven databases, including Nr, Nt, Pfam, KOG/COG, Swiss-Prot, KEGG and GO, were used to annotate the functions of all unigenes. A total of 15,969 unigenes (97.5%) were annotated from at least one database, while 8,154 annotated unigenes were found in all databases (Figure 3A). For GO analysis, “metabolic process” (5,779 unigenes) showed the most enrichment pathways in biological process. “Cell” and “Binding” represented the major groups in the category of cellular component and molecular function, respectively (Figure 3B). For KOG categorization, “general function prediction only” (2,055 unigenes) was the largest among 26 functional groups, followed by “signal transduction mechanisms” and “post-translational modification, protein turnover, chaperones” (Figure 3C). A total of 442 unigenes were classified in the “secondary metabolites biosynthesis, transport and catabolism” group. For KEGG classification, the metabolism group exhibited the most genes (Figure 3D). In this group, 168 unigenes were found to represent “xenobiotics biodegradation and metabolism.” Only 376 unigenes were related to secondary metabolism, including a large number of genes (22.9%) associated with terpenoid metabolism (Supplementary Table 3).

### Identification of lncRNAs

lncRNAs are key functional regulators in plant biological processes (Long et al., 2017). In this study, 569, 1,324, 3,001, and 2,223 candidate lncRNAs were identified by the Coding-Non-Coding-Index (CNCI), Coding Potential Calculator (CPC), Pfam, and PLEK databases, respectively. A total of 4,678 lncRNAs were identified from at least one database. The full lengths ranged from 1,500 to 4,000 bp, with an average length of 2,656 bp (Supplementary Figure 2).

### Comparative Analysis of DEGs

**FIGURE 4:**
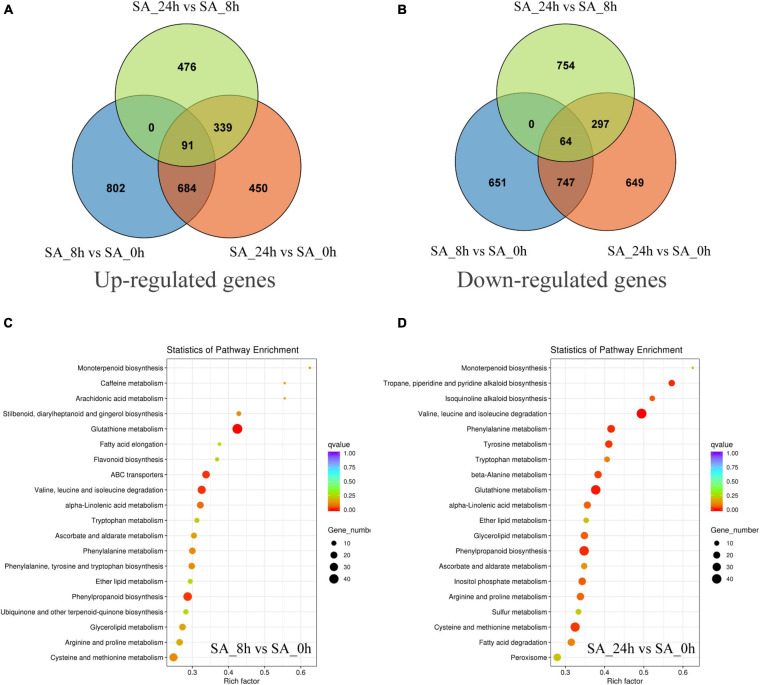
The distribution and classification of differentially expressed genes under SA treatments. **(A,B)** Venn figures represented the numbers of up-regulated **(A)** and down-regulated **(B)** genes in three comparison types (“SA_8h vs. SA_0h,” “SA_24h vs. SA_0h,” and “SA_24h vs. SA_8h”). **(C,D)** The pathway enrichment analysis of the up-regulated genes involved in “SA_8h vs. SA_0h” **(C)** or “SA_24h vs. SA_0h” **(D)** based on KEGG databases.

To investigate transcript changes in PTHRCs after SA treatments, DEG profiles were determined using the FPKM mapped reads method. The DEG numbers of three comparisons (“SA_8h vs. SA_0h,” “SA_24h vs. SA_0h” and “SA_24h vs. SA_8h”) are shown in Figures 4A,B. Compared to controls, a total of 775 up-regulated and 811 down-regulated unigenes were, respectively, observed after 8 and 24 h of SA treatments in PTHRCs. Only 91 or 64 genes were up- or down-regulated in all three comparisons, respectively. The up-regulated genes involved in “SA_8h vs. SA_0h” or “SA_24h vs. SA_0h” were further assigned in KEGG pathway analysis (Figures 4C,D). We found a series of up-regulated genes related to secondary metabolism, including terpenoid, flavonoid, phenylpropanoid and glutathione. Notably, the pathway of “monoterpenoid biosynthesis” reached the peak of factors among all KEGG pathways in both comparisons.

### DEGs Involved in the SA Signaling Network

**FIGURE 5:**
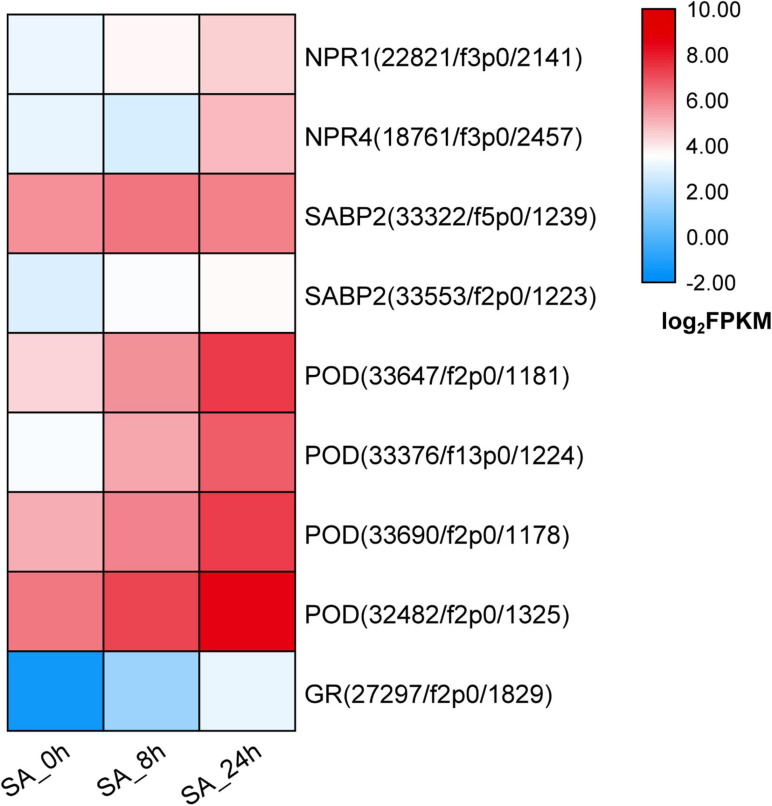
Expression of candidate genes involved in SA signaling network. The heat graph shows the up-regulated genes encoding SA-binding proteins and reactive oxygen species (ROS) scavenging enzymes under SA treatments. The fragment per kilobase per million (FPKM) for unigenes were log_2_ transformed.

To illuminate how the SA signaling network functions in *P. tunicoides*, DEGs involved in SA perception or transduction pathways were analyzed based on transcriptome data. In PTHRCs, exogenous application of SA increased the expression levels of two *NPR* genes, named *PtNPR1* (22,821/f3p0/2,141) and *PtNPR4* (18,761/f3p0/2,457), which are widely reported as SA-binding proteins in model plants (Innes, 2018; Figure 5). SA-binding protein 2 (*SABP2*) was also up-regulated after SA elicitation (Figure 5). Furthermore, a series of genes encoding reactive oxygen species (ROS) scavenging enzymes were found in the transcriptome, including *PODs* and *GRs*. SA continuously up-regulated four *PtPODs* and one *PtGR*, as well as promoting enzyme activities of POD and GR at 8 h elicitation (Figure 5 and Supplementary Figure 3).

### Transcription Factors (TFs) Involved in Saponin Biosynthesis

**FIGURE 6:**
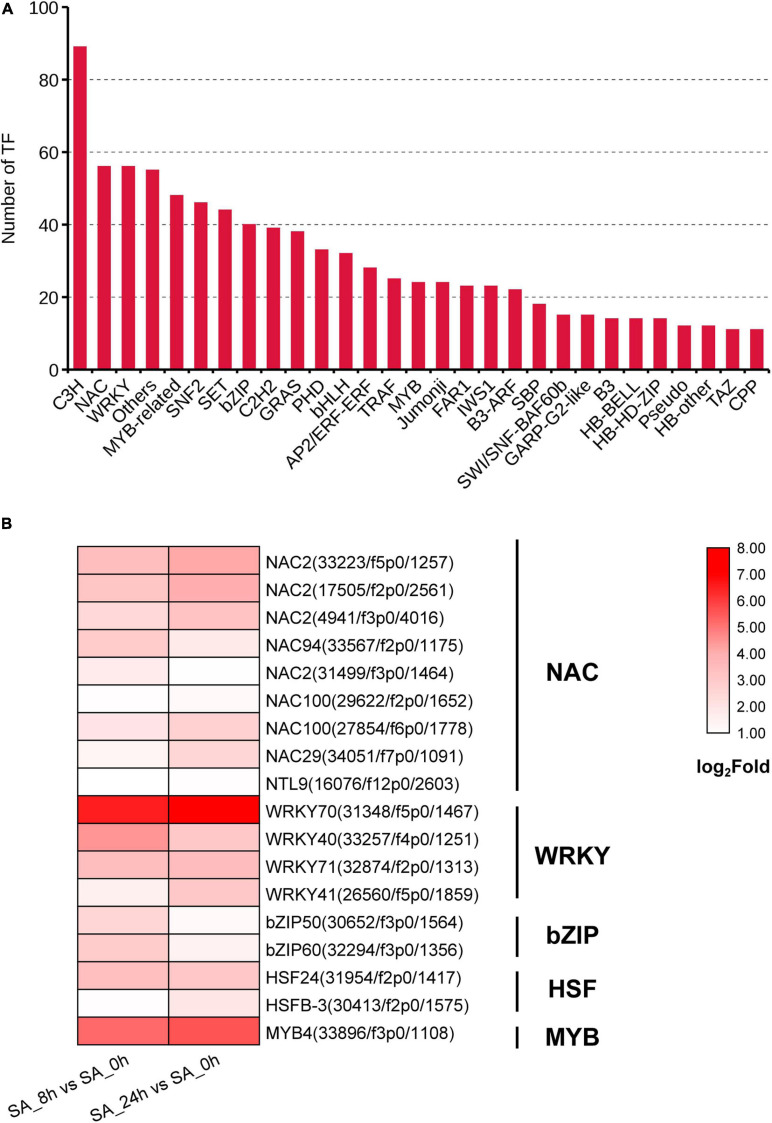
Identification of transcription factor (TF) families. **(A)** The unigene numbers annotated to TF families. **(B)** The heat graph shows the up-regulated TF genes belong to NAC, WRKY, bZIP, HSF and MYB families under SA treatments. The fold changes for two comparison types (SA_8h vs. SA_0h and SA_24h vs. SA_0h) were log_2_ transformed.

A total of 1,129 TF unigenes from 29 families were found in the PTHRC transcriptome, and the three largest TF families were related to C3H (89), NAC (56) and WRKY (56) (Figure 6A). Compared to controls, 275 or 300 DEGs associated with TFs were induced after 8 h or 24 h of SA treatments, respectively. Among them, 42 DEGs were continuously up-regulated with SA elicitation in both 8 and 24 h time points, of which several TF families generally participate in stress response and secondary metabolism (Endt et al., 2002), such as NAC (9), WRKY (4), bZIP (2), HSF (2), and MYB (1) families (Figure 6B). In particular, SA increased gene expression of *PtWRKY70* (31,348/f5p0/1,467), *PtMYB4* (33,896/f3p0/1,108), and *PtWRKY40* (33,257/f4p0/1,251), suggesting that these unigenes might participate in SA-elicited secondary metabolism.

### Candidate Genes Involved in Triterpenoid Saponin Biosynthesis

**FIGURE 7:**
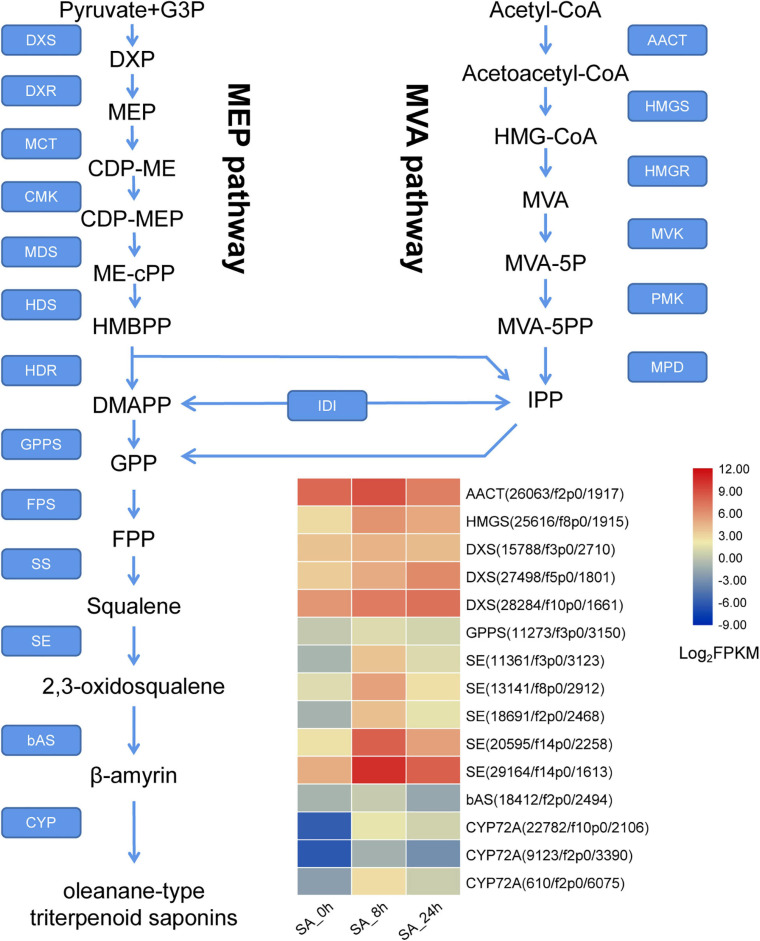
The putative expression profiles of SA-elicited saponin biosynthesis in *P. tunicoides* hairy root cultures. The heat graph shows the up-regulated unigenes encoding enzymes involved in saponin biosynthesis under SA treatments. The FPKM values of unigenes were log2 transformed.

In the PTHRC transcriptome, six unigenes were identified as putative genes in the MVA pathway, including one *AACT* (acetyl-CoA acyltransferase), one *HMGS* (3-hydroxy-3-methylglutaryl-CoA synthase), two *HMGRs*, one *PMK* (phosphomevalonate kinase) and one *MVD* (mevalonate 5-diphosphate decarboxylase). In addition, 17 putative unigenes were recognized in the MEP pathway, containing eight *DXSs* (1-deoxy-D-xylulose-5-phosphate synthase), two *DXRs* (1-deoxy-D-xylulose 5-phosphate reductoisomerase), one *MDS* (2-C-methyl-D-erythritol 2,4-cyclodiphosphate synthase), four *HDSs* (4-hydroxy-3-methylbut-2-en-1-yl diphosphate synthase) and two *HDRs* (4-hydroxy-3-methylbut-2-enyl diphosphate reductase). Furthermore, 31 unigenes were putatively related to formation of β-amyrin, including one *GPPS* (geranyl diphosphate synthase), two *FPSs* (farnesyl pyrophosphate synthase), one *SS* (squalene synthase), 22 *SEs* (squalene epoxidase) and six *bASs* (β-amyrin synthase). Information for all of these genes is present in Figure 7 and Supplementary Table 4. Among them, a total of 35 DEGs were found after SA treatments in PTHRCs. We observed that SA significantly promoted the expression levels of one *AACT*, one *HMGS* and three *DXSs* (Figure 7). These enzyme genes were all located at the early stage of MVA and MEP pathways. Moreover, unigenes including one *GPPS*, 16 *SE* and one *bAS* were rapidly up-regulated at 8 h of SA treatment. Five *SEs* showed at least 10-fold up-regulation compared to control (Figure 7), suggesting that these *PtSE* genes might be the key regulatory factors in response to SA elicitation in PTHRCs.

**FIGURE 8:**
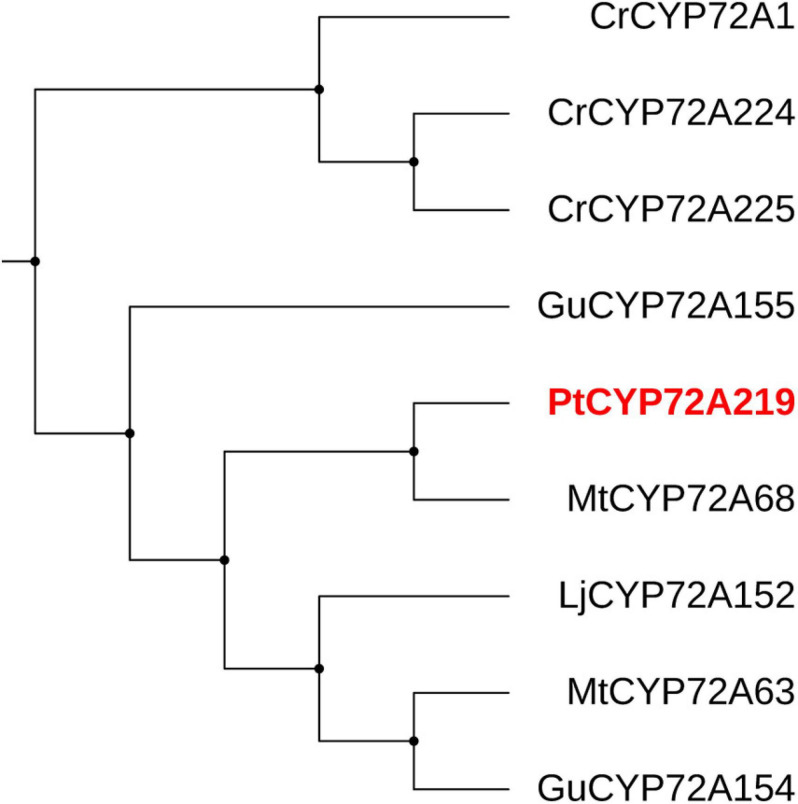
The phylogenetic tree analysis between PtCYP72A219 and eight known CYP72As participated in terpenoid metabolism. The CYP72As from different species were obtained from UniProt database: CrCYP72A1 (No. Q05047), CrCYP72A224 (No. U5NE19), CrCYP72A225 (No. W8JWW3), GuCYP72A154 (No. H1A988), GuCYP72A155 (No. H1A989), MtCYP72A63 (No. H1A981), MtCYP72A68 (No. Q2MJ19), LjCYP72A152 (No. H1A986).

Cytochrome P450s (CYPs) function in site-specific oxidization of the oleanane skeleton (Banerjee and Hamberger, 2018). A total of 114 putative *CYP* unigenes were recognized in transcriptome data, which belong to diverse CYP subfamilies like CYP71A, CYP72A, CYP716A, CYP89A, and CYP94A. Given that CYP72A and CYP716A subfamilies are primarily involved in the biosynthesis of triterpenoid saponin diversification (Ghosh, 2017), we found 29 genes belonging to CYP72A subfamilies, while only two genes belonged to CYP716A families (Supplementary Table 4). In total, 21 CYP72A genes were significantly up-regulated after 8 h of SA applications, and *PtCYP72A219* (22,782/f10p0/2,106) showed the largest increase compared to controls (206.8-folds) (Figure 7 and Supplementary Table 4). Phylogenetic trees were inferred for PtCYP72A219 and eight known CYP72As that participated in terpenoid metabolism, and PtCYP72A219 showed the highest similarity (54.7%) to *MtCYP72A58* in *Medicago truncatula* (Figure 8).

### qRT-PCR Validation of DEGs

**FIGURE 9:**
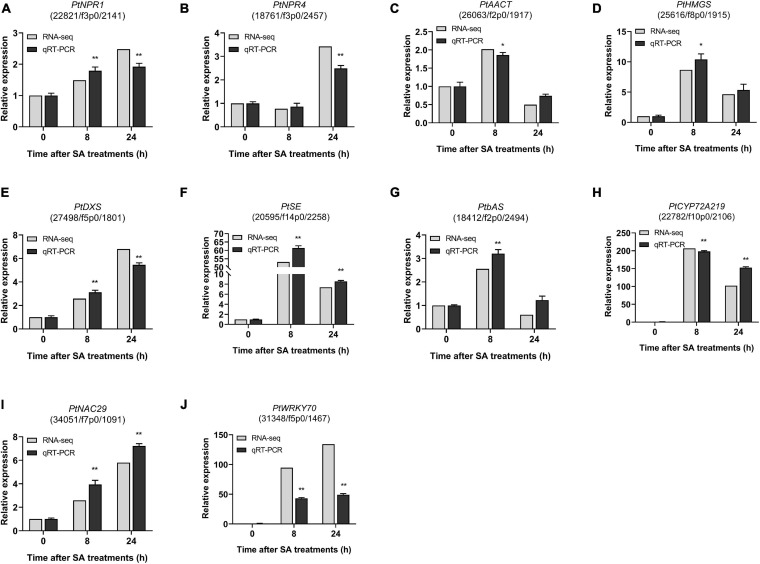
qRT-PCR of candidate genes involved in SA-elicited saponin biosynthesis. The relative expression of ten genes from RNA-seq and qRT-PCR under 0, 8, 24 h of SA treatments. **(A)** *PtNPR1*, **(B)** *PtNPR4*, **(C)** *PtAACT*, **(D)** *PtHMGS*, **(E)** *PtDXS*, **(F)** *PtSE*, **(G)** *PtbAS*, **(H)** *PtCYP219*, **(I)** *PtNAC29*, and **(J)** *PtWRKY70*. The values represent the mean value ± SE from three biological replicates. *, ** Indicate significant differences in comparison with values of SA_0 h at *P* < 0.05 and *P* < 0.01 level (*t*-test), respectively.

To validate the RNA-Seq transcriptome results, 10 candidate DEGs involved in the SA signaling network and saponin biosynthesis in PTHRCs were selected for measurement of transcript levels by qRT-PCR analysis, including *PtNPR1*, *PtNPR4*, *PtAACT*, *PtHMGS*, *PtDXS*, *PtSE*, *PtbAS*, *PtCYP72A219*, *PtNAC29*, and *PtWRKY70*. The fold change of qRT-PCR and RNA-seq data were compared. As shown in Figure 9, the relative expression results of these 10 candidate DEGs were generally consistent with the RNA-seq values. In particular, the expression levels of *PtSE*, *PtbAS*, *PtCYP72A219*, *PtNAC29*, and *PtWRKY70* increased significantly at the early stage of SA treatments.

## Discussion

As sequencing technologies continue to develop, the PacBio SMRT platform is becoming increasingly popular for full-length sequencing applications in secondary metabolism. For instance, 20.37% of FLNC transcripts obtained from SMRT sequencing were putatively novel genes in pitaya fruit, and six novel genes that might be involved in betalain biosynthesis were identified (Wu et al., 2020). Herein, we performed the first full-length transcriptome analysis of *P. tunicoides* using a combination of Illumina RNA-seq and PacBio SMRT sequencing. A total of 430,117 CCS reads were obtained with SMRT, containing 35,262 non-redundant transcripts (Figure 2). The proportion of long transcript reads and full-length genes from SMRT sequencing were much higher than those of Illumina RNA-seq analysis (Table 1), which was consistent with other reports (Chen et al., 2019; Wu et al., 2020), indicating that SMRT has a prominent advantage in gene length and CDS completeness prediction. Based on the long transcript reads and low error rate, 97.5% of unigenes were successfully annotated from at least one database (Figure 3A). GO analysis revealed that “metabolic process” had the most enriched pathways in biological process (Figure 3B), while KEGG annotation showed 22.9% of genes in secondary metabolism associated with terpenoid metabolism (Supplementary Table 3), suggesting abundant genes were involved in primary and secondary metabolism, and the majority of pathways of secondary metabolism were related to terpenoid biosynthesis in *P. tunicoides*.

SA acts as a critical regulator to modulate hypersensitive response to biotic and abiotic stresses (Lu et al., 2016). Few transcriptome studies have been reported on the relationship between SA-elicited terpenoid biosynthesis and the molecular network. Ye et al. (2020) found that exogenous application of SA enhanced terpene trilactone levels in *Ginkgo biloba* leaf; RNA-seq showed 249 DEGs between SA treatment and control, containing candidate structural genes (*HMGR*, *CYP450*) and TFs (*MYB* and *WRKY*) involved in terpene synthesis (Ye et al., 2020). Herein, the gene numbers and pathway enrichments of DEGs were respectively obtained from five divided groups: “SMRT+RNA-seq,” “single RNA-seq,” “overlap,” “unique in SMRT” and “unique in RNA-seq” (Supplementary Table 5). We noticed these five groups all exhibited the same tendency in comparison of up-regulated and down-regulated gene numbers under 8 or 24 h of SA treatments compared with the control (Supplementary Table 5). Besides, the enriched GO and KEGG terms of DEGs from both “SMRT+RNA-seq” and “single RNA-seq” were essentially the same in “SA_24 h vs. SA_0 h,” while different in “SA_8 h vs. SA_0 h” (Supplementary Table 5). These results indicated that the pathway enrichment results might be distinctively analyzed by next- or third-generation sequencing platforms at different stages of SA treatments. KEGG analysis of the up-regulated DEGs revealed that “terpenoid biosynthesis” was the highest among all pathways (Figures 4C,D), indicating that SA enhanced the expression of terpenoid metabolism in PTHRCs. Additionally, we noticed several DEGs were enriched in the biosynthesis pathway of other secondary metabolites, including phenylpropanoid, flavonoid and isoquinoline alkaloid (Supplementary Table 3) consistent with Shan et al. (2020), suggesting SA might influence multiple processes of secondary metabolism in hairy roots.

NPRs were recently highlighted as the receptor of SA; NPR1 functions positively in SA perception, while NPR3/4 has negative roles in *Arabidopsis* (Innes, 2018). Here, the expression levels of both *PtNPR1* and *PtNPR4* increased with SA elicitation, suggesting that NPR-related perception might also exist in *P. tunicoides.* Furthermore, SA is also known to exhibit crosstalk with ROS signaling pathways (Tewari and Paek, 2011); endogenous ROS levels were regulated by diverse antioxidant enzymes, such as superoxide dismutase (SOD), catalase (CAT), POD, and GR (Zhou et al., 2017; Buraphaka and Putalun, 2020). The POD and CAT activities were up-regulated in SA-elicited triterpenoid synthesis in *Centella* leaves (Buraphaka and Putalun, 2020); the involvement of nitric oxide (NO) in SA-induced ginsenoside accumulation might be mediated by multiple antioxidant enzymes (Tewari and Paek, 2011). In this study, we found that both transcript profiles and enzyme activities of POD and GR were continuously enhanced after SA treatments, which indicates that ROS might participate in the SA signal cascade in PTHRCs.

As the rate-limiting enzymes in triterpenoid skeleton biosynthesis, AACT and DXS represent the initial steps in MVA and MEP pathways, while SE and bAS catalyze the last two steps in oxidation and cyclization of squalene to β-amyrin (Moses et al., 2014). Previous studies showed that SA stimulated the expression of these key enzyme genes, thus resulting in terpenoid accumulation in plants (Pu et al., 2009; Wu et al., 2020). Here, numerous genes encoding these enzymes were up-regulated in response to SA in PTHRCs (Figure 7). We observed that PtSEs showed the highest gene numbers associated with formation of the triterpenoid skeleton, and most *PtSEs* were significantly up-regulated after SA treatments in PTHRCs (Supplementary Table 4). Previous studies illuminated that SA stimulated the gene expression of *SE* in *Withania somnifera* (Kushwaha et al., 2019) and *Nigella sativa* (Elyasi et al., 2016), and overexpression of *PgSE1* significantly increased ginsenoside production in transgenic roots (Han et al., 2020). Therefore, SA triggers the enzymatic network involved in saponin biosynthesis, and the oxidation of squalene might be the key step in these processes.

CYP450s represent one of the largest enzymatic families, and are essential in terpenoid skeleton diversification (Banerjee and Hamberger, 2018). Members of the CYP72A subfamily are involved in oleanolic acid-derived saponin biosynthesis (Seki et al., 2015). MtCYP72A68 catalyzes the carboxylation of oleanolic acid at the C-23 position to form gypsogenic acid in *Medicago truncatula* (Tzin et al., 2019), while GuCYP72A154 oxidizes at the C-30 position of 11-oxo-β-amyrin to produce glycyrrhetinic acid in *Glycyrrhiza uralensis* (Seki et al., 2011). Li et al. (2017) reported that *Meyerozyma guilliermondii* elicitation of *G. uralensis* increased glycyrrhizin acid by enhancing *CYP72A154* expression and endogenous SA contents. We also observed a series of SA-elicited genes from the CYP72A family in PTHRCs, with a high similarity between PtCYP72A219 and MtCYP72A68, suggesting that PtCYP72A219 likely participates in the SA-elicited saponin biosynthesis pathway.

TF families have essential roles in transcriptional regulation of secondary metabolism through modulating downstream gene expression associated with terpenoid biosynthetic enzymes (Endt et al., 2002). PgWRKY4X positively regulates ginsenoside biosynthesis via binding to a W-box element in the promoter of *PgSE* (Yao et al., 2020), while AaNAC2/3/4 binds to a 28-bp fragment of the NAC binding site (NACBS) in the *AaTPS1* promoter to accumulate the kiwifruit monoterpene volatiles (Nieuwenhuizen et al., 2015). Here, we observed that a series of TF genes from the WRKY and NAC families were up-regulated after SA treatments, and expression of *PtWRKY70* (31,348/f5p0/1,467) showed the largest increase. Interestingly, we also found W-box and NACBS *cis*-elements in promoters of SA-elicited genes containing *AACT*, *HMGS*, *DXS*, *SE*, and *CYP72A* based on full-length transcriptome data (Supplementary Table 6), suggesting a possible interactive relationship between TFs and key enzyme genes involved in SA-elicited saponin metabolism in *P. tunicoides.*

**FIGURE 10:**
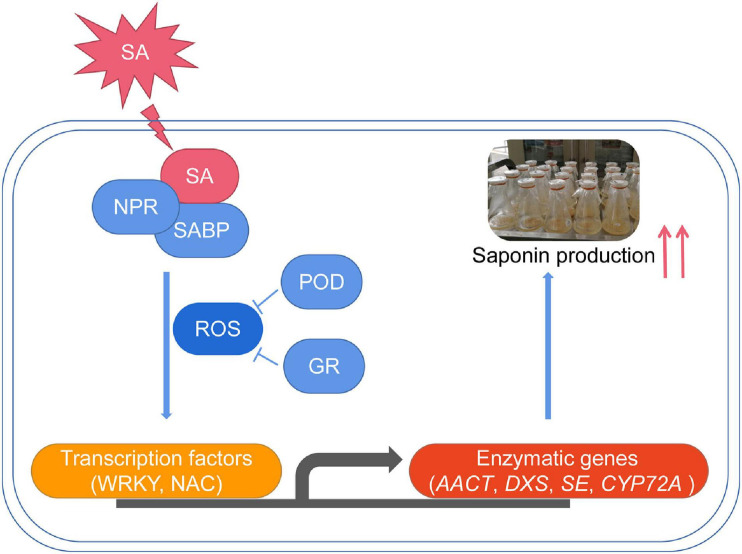
The proposed transcriptional regulatory network for SA-elicited saponin biosynthesis in *P. tunicoides* hairy root cultures.

In summary, we concluded that SA significantly increased the triterpenoid saponin accumulation in *P. tunicoides*. The first full-length transcriptome of *P. tunicoides* was produced, providing a prominent advantage in gene length and CDS completeness prediction. DEGs related to SA-elicited saponin biosynthesis were identified on the transcriptome. Finally, a putative regulatory chain was illustrated, involving elicitor perception (NPRs), signaling cascade (ROS), TF regulation (WRKY, NAC) and enzymatic catalysis (*AACT*, *DXS*, *SE*, *CYP72A*) (Figure 10). These results improve the comprehensive understanding of the SA-elicited triterpenoid saponin biosynthesis pathway, and provide guidance for improving saponin production of *P. tunicoides*.

## Data Availability Statement

The datasets presented in this study can be found in online repositories. The names of the repository/repositories and accession number(s) can be found below: https://bigd.big.ac.cn/gsa/, CRA002795.

## Author Contributions

LS and ZZ designed the whole project. HQ and SL performed the experiments. HW provided the plant materials. CW, CH, and MX provided assistance in performing the experiments. LS wrote the manuscript. ZZ reviewed the manuscript. All authors contributed to the article and approved the submitted version.

## Conflict of Interest

The authors declare that the research was conducted in the absence of any commercial or financial relationships that could be construed as a potential conflict of interest.

## Footnotes

1 https://bigd.big.ac.cn/gsa/

2 ftp://ftp.ncbi.nlm.nih.gov/blast/db

3 http://pfam.xfam.org

4 http://www.ncbi.nlm.nih.gov/KOG

5 http://ftp.ebi.ac.uk/pub/databases/swissprot

6 http://www.genome.jp/kegg

7 http://geneontology.org

8 http://www.ebi.ac.uk/Tools/hmmer/search/hmmscan

9 https://github.com/PacificBiosciences/ANGEL

10 https://itol.embl.de

11 https://www.uniprot.org

## Supplementary Material

Supplementary Figure 1 HPLC analysis of two representative saponin aglycones in *P. tunicoides* hairy root extracts with SA elicitation.

Supplementary Figure 2 Length distribution of lncRNAs and mRNAs.

Supplementary Figure 3 Effects of SA on antioxidant enzyme activities.

Supplementary Table 1 Sequence of primer pairs used in qRT-PCR analysis.

Supplementary Table 2 Summary of the transcriptome data from Illumina platform.

Supplementary Table 3 Summary of genes related to secondary metabolism based on KEGG annotation.

Supplementary Table 4 Summary of putative unigenes encoding enzymes involved in triterpenoid saponin biosynthesis based on transcriptome data.

Supplementary Table 5 Summary of the gene numbers and pathway enrichments of DEGs, respectively, obtained from five divided groups.

Supplementary Table 6 Information of NACBS and W-box *cis*-elements in predicted promoters of candidate genes based on transcriptome data.
