# Supplementary material for: Full-Length Transcriptome Analyses of Genes Involved in Triterpenoid Saponin Biosynthesis of Psammosilene tunicoides Hairy Root Cultures With Exogenous Salicylic Acid
Source: Front Genet. 2021 Mar 29;12:657060. doi: 10.3389/fgene.2021.657060 (PMC8039526; doi:10.3389/fgene.2021.657060)
Supplement: Supplementary Figure 1 — HPLC analysis of two representative saponin aglycones in P. tunicoides hairy root extracts with SA elicitation. [file Image_1.PDF]

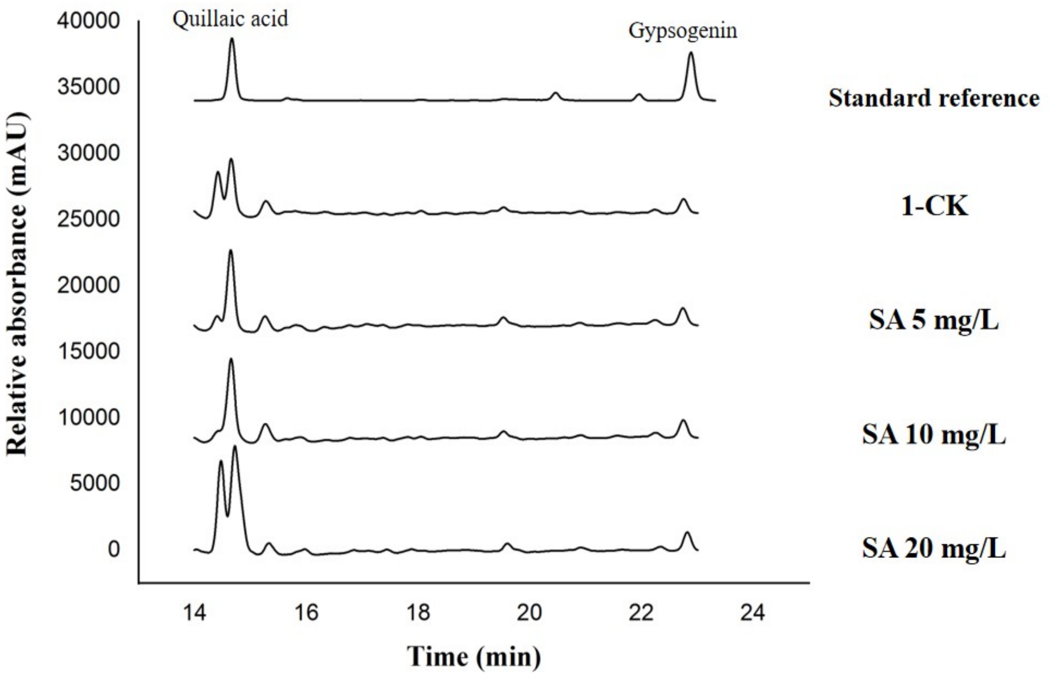

Supplementary Fig. S1 HPLC analysis of two representative saponin aglycones in *P. tunicoides* hairy root extracts with SA elicitation. The identification and qualification of quillaic acid and gypsogenin in control (1-CK) and PTHRCs elicited by 5, 10 and 20 mg/L SA for 1 day.

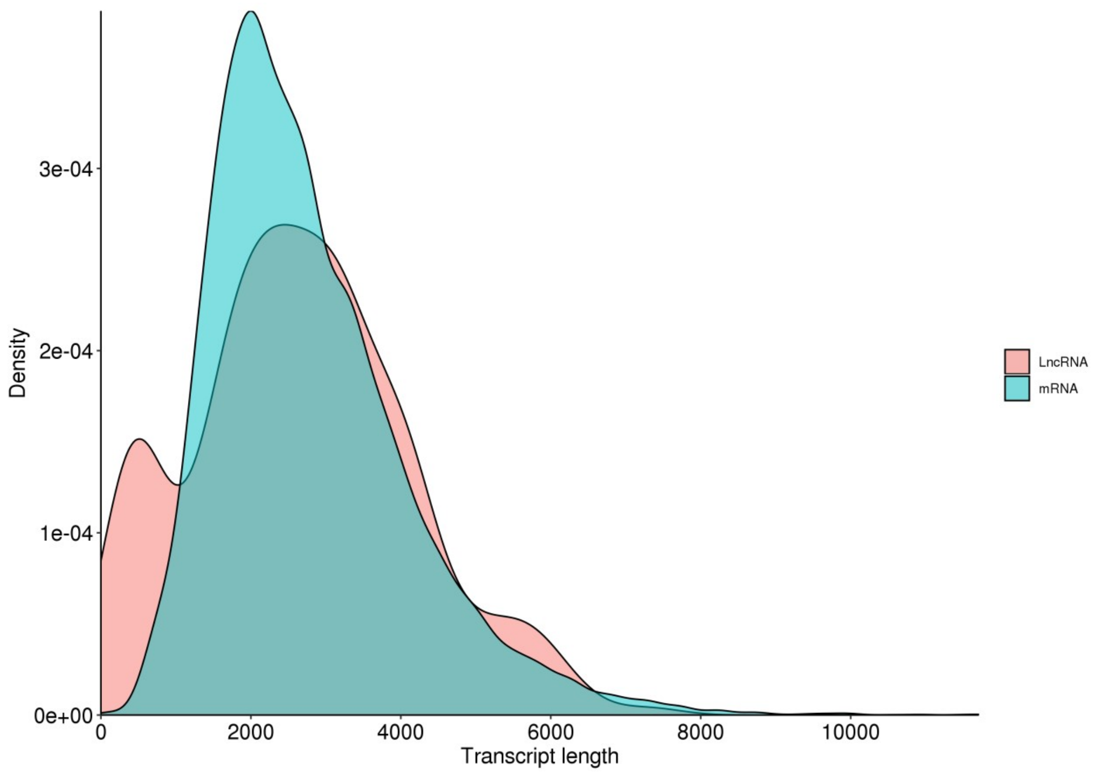

Supplementary Fig. S2 Length distribution of lncRNAs and mRNAs.

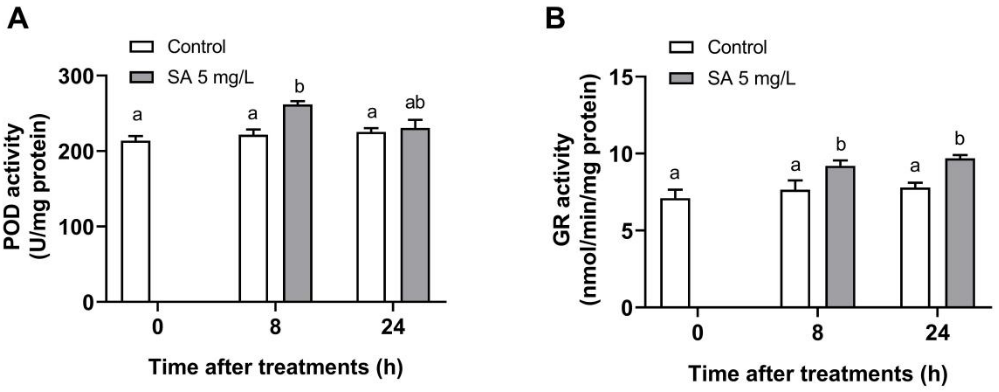

Supplementary Fig. S3 Effects of SA on antioxidant enzyme activities. The POD(A) and GR(B) activities of *P. tunicoides* hairy roots treated with 5 mg/L SA and controls. Data are means ± SD. Significant differences ($P \leqslant 0.05$) are indicated by different letters.
