# Supplementary material for: Full-Length Transcriptome Analyses of Genes Involved in Triterpenoid Saponin Biosynthesis of Psammosilene tunicoides Hairy Root Cultures With Exogenous Salicylic Acid
Source: Front Genet. 2021 Mar 29;12:657060. doi: 10.3389/fgene.2021.657060 (PMC8039526; doi:10.3389/fgene.2021.657060)
Supplement: Supplementary Table 1 — Sequence of primer pairs used in qRT-PCR analysis. [file Data_Sheet_1.docx]

Supplementary Material

**Supplementary Table S1 Sequence of primer pairs used in qRT-PCR analysis.**

| **Name** | **Orientation** | **Sequence (5'-3')** |
| --- | --- | --- |
| *PtNPR1* | Forward | CCATTGTGATGCCAAGACCA |
|  | Reverse | TTCCTCATAGCTGCAACGTGTAA |
| *PtNPR4* | Forward | ACAGTACCAGGAGGGAACAACC |
|  | Reverse | AAGCTGCTGCAAAAGTTAGGG |
| *PtAACT* | Forward | TGGTGCATTTTCATGGGAGA |
|  | Reverse | CAACTTGGCAGGGTCATACTTACT |
| *PtHMGS* | Forward | ATCTTGCTACGGTTCTTGCTTCT |
|  | Reverse | GCCTGATCTTCGTGCCATCT |
| *PtDXS* | Forward | TGCCGCAGTAATGGAGGTG |
|  | Reverse | GGATTGTATTGTAGACAGCAAGAGC |
| *PtSE* | Forward | CACCCTATGACCCCAGACCTT |
|  | Reverse | GCACTGAACCCACAAAATAACCT |
| *PtbAS* | Forward | AGCCAGTCAAACAGTCACAGCA |
|  | Reverse | CACCCATGAAACATCATACCCA |
| *PtCYP72A219* | Forward | GCAATAAAGGGAGGTGAAACAAC |
|  | Reverse | TCAACGTCCAAACAAGCAAAGT |
| *PtNAC29* | Forward | ATGCCTCGTTTCTATTATTCCTGA |
|  | Reverse | ACTTCCTGTCTCGTGGGGTG |
| *PtWRKY70* | Forward | TGGAAGAGTTGGAAAGAGGCA |
|  | Reverse | AACAAAGGACGATTTAGTAGGAGACT |
| *PtActin* | Forward | TTCGAGCAGGAATCTGACACAT |
|  | Reverse | CAATGATGGCTGGAAGAGGAC |

**Supplementary Table. S2 Summary of the transcriptome data from Illumina platform**

| **Group** | **Sample** | **Raw Reads** | **Clean Reads** | **Clean Bases (Gb)** | **Q20(%)** | **Q30(%)** | **GC(%)** |
| --- | --- | --- | --- | --- | --- | --- | --- |
| SA_0h | SA_0h_1 | 62376684 | 61105726 | 9.17 | 97 | 91.81 | 43.04 |
|  | SA_0h_2 | 54215458 | 53402878 | 8.01 | 97.43 | 92.72 | 43.08 |
|  | SA_0h_3 | 60207566 | 59204994 | 8.88 | 96.08 | 89.79 | 43.05 |
| SA_8h | SA_8h_1 | 50470002 | 49118248 | 7.37 | 97.61 | 93.1 | 43 |
|  | SA_8h_2 | 52260000 | 50981334 | 7.65 | 97.38 | 92.56 | 42.3 |
|  | SA_8h_3 | 55468902 | 53915408 | 8.09 | 97.2 | 92.16 | 44.05 |
| SA_24h | SA_24h_1 | 53568466 | 52323930 | 7.85 | 97.18 | 92.09 | 42.67 |
|  | SA_24h_2 | 48529288 | 47256528 | 7.09 | 97.4 | 92.6 | 42.72 |
|  | SA_24h_3 | 50594978 | 48225558 | 7.23 | 97.49 | 92.82 | 42.45 |
| Total |  | 487691344 | 475534604 | 71.34 |  |  |  |

**Supplementary Table. S3 Summary of** **genes related to** **secondary metabolism based on KEGG annotation**

| Pathway ID | KEGG defination | Total  gene number | DEG number in  ‘SA_8h vs SA_0h’ | DEG number in  ‘SA_24h vs SA_0h’ |
| --- | --- | --- | --- | --- |
| *Terpenoid metabolism* | |  |  |  |
| ko00905 | Brassinosteroid biosynthesis | 8 | 6 | 6 |
| ko00906 | Carotenoid biosynthesis | 17 | 10 | 8 |
| ko00904 | Diterpenoid biosynthesis | 1 | 0 | 0 |
| ko00903 | Limonene and pinene degradation | 11 | 4 | 8 |
| ko00902 | Monoterpenoid biosynthesis | 8 | 5 | 7 |
| ko00909 | Sesquiterpenoid and triterpenoid biosynthesis | 9 | 3 | 6 |
| ko00900 | Terpenoid backbone biosynthesis | 32 | 6 | 12 |
| *Other metabolism* | |  |  |  |
| ko00940 | Phenylpropanoid biosynthesis | 115 | 54 | 70 |
| ko00945 | Stilbenoid, diarylheptanoid and gingerol biosynthesis | 21 | 17 | 14 |
| ko00960 | Tropane, piperidine and pyridine alkaloid biosynthesis | 28 | 11 | 22 |
| ko00941 | Flavonoid biosynthesis | 19 | 15 | 12 |
| ko00950 | Isoquinoline alkaloid biosynthesis | 23 | 10 | 18 |
| ko00232 | Caffeine metabolism | 9 | 7 | 6 |
| ko00261 | Monobactam biosynthesis | 6 | 2 | 3 |
| ko00908 | Zeatin biosynthesis | 8 | 4 | 4 |
| ko00254 | Aflatoxin biosynthesis | 1 | 0 | 0 |
| ko00965 | Betalain biosynthesis | 2 | 0 | 0 |
| ko00966 | Glucosinolate biosynthesis | 4 | 0 | 0 |
| ko00901 | Indole alkaloid biosynthesis | 4 | 0 | 0 |
| ko00524 | Neomycin, kanamycin and gentamicin biosynthesis | 15 | 0 | 0 |
| ko00401 | Novobiocin biosynthesis | 1 | 0 | 0 |
| ko00521 | Streptomycin biosynthesis | 20 | 0 | 0 |
| ko01051 | Biosynthesis of ansamycins | 2 | 0 | 0 |
| ko00281 | Geraniol degradation | 2 | 0 | 0 |
| ko00981 | Insect hormone biosynthesis | 9 | 0 | 0 |
| ko00523 | Polyketide sugar unit biosynthesis | 1 | 0 | 0 |

**Supplementary Table. S4 Summary of putative unigenes encoding enzymes involved in triterpenoid saponin biosynthesis based on transcriptome data**

| Enzyme name | Gene ID | FPKM(SA_0h) | FPKM(SA_8h) | FPKM(SA_24h） |
| --- | --- | --- | --- | --- |
| *MVA pathway* |  |  |  |  |
| AACT | 26063/f2p0/1917 | 238.83 | 482.45 | 119.05 |
|  | 27274/f3p0/1822 | 45.04 | 42.48 | 50.80 |
| HMGS | 25616/f8p0/1915 | 7.07 | 61.30 | 32.75 |
|  | 25528/f5p0/1913 | 37.34 | 24.96 | 22.33 |
|  | 25931/f6p0/1898 | 100.89 | 75.77 | 48.52 |
| HMGR | 22066/f12p0/2176 | 102.69 | 73.18 | 49.63 |
|  | 23215/f4p0/2096 | 84.69 | 44.33 | 46.74 |
|  | 30594/f3p0/1561 | 118.10 | 69.93 | 41.53 |
| PMK | 23144/f2p0/2123 | 44.57 | 28.95 | 23.41 |
| MVD | 30594/f3p0/1561 | 118.10 | 69.93 | 41.53 |
| *MEP pathway* |  |  |  |  |
| DXS | 15788/f3p0/2710 | 14.55 | 23.19 | 18.20 |
|  | 27498/f5p0/1801 | 10.94 | 29.65 | 83.89 |
|  | 28284/f10p0/1661 | 56.31 | 130.80 | 177.28 |
|  | 16555/f5p0/2577 | 45.74 | 24.06 | 63.30 |
|  | 27182/f10p0/1744 | 45.07 | 26.17 | 40.47 |
|  | 29219/f3p0/1670 | 25.18 | 0.00 | 5.05 |
|  | 29505/f3p0/1639 | 258.53 | 193.30 | 151.01 |
|  | 30838/f3p0/1539 | 45.55 | 32.49 | 33.03 |
| DXR | 21561/f2p0/2238 | 8.00 | 5.60 | 9.39 |
|  | 21595/f2p0/2237 | 0.34 | 0.20 | 0.31 |
| MDS | 10041/f5p0/3254 | 12.70 | 13.13 | 9.37 |
| HDS | 11626/f2p0/3134 | 6.39 | 5.17 | 7.41 |
|  | 16687/f21p0/2575 | 15.42 | 9.30 | 16.92 |
|  | 25875/f13p0/1866 | 19.54 | 23.88 | 21.45 |
|  | 26419/f3p0/1885 | 18.00 | 9.42 | 6.15 |
| HDR | 28938/f5p0/1670 | 46.56 | 31.99 | 50.18 |
|  | 29138/f3p0/1630 | 62.90 | 37.91 | 47.75 |
| *IPP to triterpenoid saponin* |  |  |  |  |
| GPPS | 11273/f3p0/3150 | 1.16 | 2.44 | 1.81 |
| FPS | 31338/f2p0/1496 | 27.74 | 23.70 | 28.28 |
|  | 32231/f3p0/1495 | 289.61 | 141.07 | 112.24 |
| SS | 29223/f3p0/1689 | 59.25 | 34.77 | 26.93 |
| SE | 11361/f3p0/3123 | 0.52 | 14.29 | 2.38 |
|  | 13141/f8p0/2912 | 2.51 | 40.09 | 5.96 |
|  | 15186/f6p0/2699 | 3.32 | 22.68 | 2.35 |
|  | 18691/f2p0/2468 | 0.49 | 16.01 | 3.16 |
|  | 19415/f2p0/2367 | 7.51 | 5.15 | 2.79 |
|  | 20595/f14p0/2258 | 5.65 | 300.26 | 41.61 |
|  | 22438/f2p0/2149 | 1.86 | 7.58 | 5.32 |
|  | 23360/f5p0/2088 | 18.63 | 14.30 | 11.85 |
|  | 25137/f2p0/1982 | 7.94 | 9.98 | 5.41 |
|  | 2524/f2p0/4676 | 0.77 | 2.73 | 1.04 |
|  | 25700/f6p0/1917 | 22.47 | 22.30 | 26.45 |
|  | 25976/f2p0/1891 | 15.68 | 20.18 | 22.47 |
|  | 28921/f7p0/1637 | 162.01 | 371.67 | 222.02 |
|  | 29164/f14p0/1613 | 29.22 | 1398.77 | 300.15 |
|  | 31579/f18p0/1413 | 41.28 | 334.01 | 144.30 |
|  | 32245/f2p0/1405 | 7.05 | 25.05 | 18.42 |
|  | 3518/f2p0/4398 | 5.28 | 4.39 | 2.31 |
|  | 3561/f3p0/4352 | 1.75 | 4.40 | 2.68 |
|  | 3690/f2p0/4271 | 1.75 | 6.50 | 4.53 |
|  | 4406/f4p0/4152 | 0.22 | 0.48 | 0.20 |
|  | 4882/f3p0/4054 | 10.21 | 58.20 | 24.39 |
|  | 7976/f2p0/3559 | 8.49 | 30.30 | 12.25 |
| bAS | 18412/f2p0/2494 | 0.51 | 1.31 | 0.31 |
|  | 17647/f2p0/2568 | 7.22 | 9.48 | 11.71 |
|  | 14467/f4p0/2777 | 46.99 | 11.17 | 22.55 |
|  | 19712/f2p0/2366 | 9.15 | 3.23 | 5.34 |
|  | 27678/f3p0/1806 | 180.47 | 42.60 | 83.37 |
|  | 29565/f2p0/1645 | 33.95 | 8.97 | 25.86 |
| CYP72A | 22782/f10p0/2106 | 0.02 | 3.45 | 1.70 |
|  | 9123/f2p0/3390 | 0.01 | 0.45 | 0.11 |
|  | 610/f2p0/6075 | 0.22 | 6.43 | 1.43 |
|  | 29230/f3p0/1661 | 2.79 | 79.00 | 12.83 |
|  | 25204/f5p0/1963 | 5.55 | 71.16 | 10.44 |
|  | 27084/f4p0/1840 | 8.73 | 109.26 | 21.81 |
|  | 22398/f2p0/2175 | 36.99 | 418.69 | 483.46 |
|  | 27513/f3p0/1795 | 37.30 | 414.85 | 109.77 |
|  | 23879/f2p0/2067 | 55.46 | 541.41 | 216.73 |
|  | 1771/f2p0/5062 | 0.06 | 0.57 | 0.89 |
|  | 2477/f2p0/4740 | 0.27 | 2.26 | 3.26 |
|  | 17779/f3p0/2536 | 2.86 | 21.99 | 13.61 |
|  | 24898/f7p0/1988 | 6.65 | 49.74 | 24.91 |
|  | 2922/f10p0/4511 | 2.32 | 16.24 | 2.64 |
|  | 7462/f4p0/3575 | 1.03 | 6.69 | 7.56 |
|  | 29243/f3p0/1756 | 24.69 | 147.41 | 48.17 |
|  | 13702/f3p0/2879 | 1.64 | 9.11 | 9.96 |
|  | 1827/f2p0/5035 | 1.53 | 6.54 | 1.32 |
|  | 2436/f2p0/4730 | 3.27 | 13.47 | 4.32 |
|  | 18412/f2p0/2494 | 0.51 | 1.31 | 0.31 |
|  | 17647/f2p0/2568 | 7.22 | 9.48 | 11.71 |
|  | 3149/f7p0/4495 | 0.20 | 0.26 | 0.01 |
|  | 1683/f2p0/4931 | 0.67 | 0.55 | 0.19 |
|  | 25412/f2p0/1988 | 17.66 | 12.30 | 15.62 |
|  | 25380/f3p0/2001 | 64.17 | 36.96 | 40.54 |
|  | 19712/f2p0/2366 | 9.15 | 3.23 | 5.34 |
|  | 23141/f2p0/2129 | 33.14 | 8.32 | 18.07 |
|  | 5416/f2p0/3951 | 0.02 | 0.00 | 0.00 |
| CYP716A | 29565/f2p0/1645 | 33.95 | 8.97 | 25.86 |
|  | 27678/f3p0/1806 | 180.47 | 42.60 | 83.37 |

**Supplementary Table. S5 Summary of the gene numbers and pathway enrichments of DEGs respectively obtained from five divided groups.**

|  |  | **SMRT**  **+RNA-seq** | **Single**  **RNA-seq** | **Overlap**  **genes** | **Unique genes in SMRT** | **Unique genes in RNA-seq** |
| --- | --- | --- | --- | --- | --- | --- |
| ***SA_8h vs SA_0h*** | |  |  |  |  | |
| Number of genes | DEGs (total) | 3,039 | 16,599 | 2,246 | 793 | 14,353 |
|  | DEGs (Up) | 1,576 | 9,971 | 1,181 | 395 | 8,790 |
|  | DEGs (Down) | 1,463 | 6,628 | 1,065 | 398 | 5,563 |
| Pathway enrichment | GO (biological process)* | Metabolic process | Oxidation-reduction process | Oxidation-reduction process | Metabolic process | Carbohydrate metabolic process |
|  | GO (molecular function)* | Catalytic activity | Cation binding | Oxidoreductase activity | Catalytic activity | Cation binding |
|  | KEGG** | Stilbenoid, diarylheptanoid and gingerol biosynthesis | Drug metabolism-cytochrome P450 | Starch,sucrose metabolism | Caffeine biosynthesis | Drug metabolism-cytochrome P450 |
| ***SA_24h vs SA_0h*** | |  |  |  |  | |
| Number of genes | DEGs (total) | 3,321 | 14,740 | 2,462 | 859 | 12,278 |
|  | DEGs (Up) | 1,564 | 7,098 | 1,147 | 417 | 5,951 |
|  | DEGs (Down) | 1,757 | 7,642 | 1,315 | 442 | 6,327 |
| Pathway enrichment | GO (biological process)* | Metabolic process | Oxidation-reduction process | Oxidation-reduction process | Metabolic process | Cellular glucan metabolic process |
|  | GO (molecular function)* | Catalytic activity | Catalytic activity | Catalytic activity | Magnesium ion binding | Heme binding |
|  | KEGG** | Nitrogen metabolism | Nitrogen metabolism | Nitrogen metabolism | Isoquinoline alkaloid biosynthesis | Drug metabolism-cytochrome P450 |

* The most enrichment pathways in GO terms (biological process or molecular function).

** The most enrichment pathways in KEGG terms.

**Supplementary Table. S6 Information of NACBS and W-box *cis*-elements in predicted promoters of candidate genes based on transcriptome data**

|  | ID | Predicted promoter length (nt) | Number of NACBS* | Number of W-box** |
| --- | --- | --- | --- | --- |
| AACT | 26063/f2p0/1917 | 267 | 3 | 1 |
| HMGS | 25616/f8p0/1915 | 70 | 0 | 1 |
| DXS | 28284/f10p0/1661 | 102 | 2 | 0 |
| SE | 11361/f3p0/3123 | 300 | 1 | 2 |
| bAS | 18412/f2p0/2494 | 192 | 1 | 0 |
| CYP72A | 22782/f10p0/2106 | 500 | 1 | 4 |

*: NAC binding sequence (NACBS): CGT[G/A]

**: W-box: TGAC
